# Modular Flow Reactors for Valorization of Kraft Lignin and Low‐Voltage Hydrogen Production

**DOI:** 10.1002/advs.202204170

**Published:** 2022-10-26

**Authors:** Se‐Jun Yim, Hyeonmyeong Oh, Yuri Choi, Gwang‐Noh Ahn, Chae‐Hyeon Park, Yong Hwan Kim, Jungki Ryu, Dong‐Pyo Kim

**Affiliations:** ^1^ Department of Chemical Engineering Pohang University of Science and Technology (POSTECH) Pohang 37673 Republic of Korea; ^2^ Department of Energy Engineering School of Energy and Chemical Engineering Ulsan National Institute of Science and Technology (UNIST) Ulsan 44919 Republic of Korea; ^3^ Emergent Hydrogen Technology R&D Center Ulsan National Institute of Science and Technology (UNIST) Ulsan 44919 Republic of Korea; ^4^ Graduate School of Carbon Neutrality Ulsan National Institute of Science and Technology (UNIST) Ulsan 44919 Republic of Korea

**Keywords:** biomass oxidation, continuous‐flow system, electron mediator, hydrogen evolution, in‐line separation

## Abstract

Recent studies have found that green hydrogen production and biomass utilization technologies can be combined to efficiently produce both hydrogen and value‐added chemicals using biomass as an electron and proton source. However, the majority of them have been limited to proof‐of‐concept demonstrations based on batch systems. Here the authors report the design of modular flow systems for the continuous depolymerization and valorization of lignin and low‐voltage hydrogen production. A redox‐active phosphomolybdic acid is used as a catalyst to depolymerize lignin with the production of aromatic compounds and extraction of electrons for hydrogen production. Individual processes for lignin depolymerization, byproduct separation, and hydrogen production with catalyst reactivation are modularized and integrated to perform the entire process in the serial flow. Consequently, this work enabled a one‐flow process from biomass conversion to hydrogen gas generation under a cyclic loop. In addition, the unique advantages of the fluidic system (i.e., effective mass and heat transfer) substantially improved the yield and efficiency, leading to hydrogen production at a higher current density (20.5 mA cm^−2^) at a lower voltage (1.5 V) without oxygen evolution. This sustainable eco‐chemical platform envisages scalable co‐production of valuable chemicals and green hydrogen for industrial purposes in an energy‐saving and safe manner.

## Graphical abstract

This study proposes an integrated modular system that can continuously produce hydrogen and value‐added chemicals by coupling lignin oxidation and water electrolysis. Due to real‐time depolymerization of lignin, byproduct separation, and catalyst recycling, hydrogen and valuable compounds can be produced more efficiently than conventional batch processes.

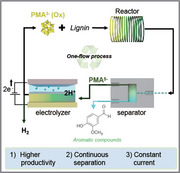

## 1 Introduction

Hydrogen is an ideal chemical that can be utilized for the decarbonization of various sectors, such as power generation, transportation, and chemical industries, in conjunction with renewable electricity generation.^[^ ^1^, ^2^, ^3^, ^4^, ^5^ ^]^ However, green hydrogen production via water electrolysis is a challenging task in terms of energy efficiency, reliability, and economics. One of the main reasons is the inherent limitation of the oxygen evolution reaction (OER), which has slow reaction kinetics and requires a large overpotential.^[^ ^6^, ^7^, ^8^, ^9^, ^10^ ^]^ For instance, the OER requires an overpotential of > 300 mV (the potential of > 1.5 V vs the reversible hydrogen electrode (RHE)) at 10 mA cm^−2^, even with expensive state‐of‐the‐art catalysts (e.g., Ru‐, and Ir‐based catalysts). Thus, water electrolysis is an energy‐intensive process that currently requires ≈50 kWh per kg hydrogen.^[^ ^11^ ^]^ Moreover, the evolved oxygen can yield serious safety concerns upon mixing with hydrogen; the volumetric ratio of hydrogen should be <4–6% to prevent ignition and explosion.^[^ ^13^, ^14^, ^15^, ^16^ ^]^ For example, biomass‐derived chemicals can be oxidatively decomposed to yield electrons and protons for low‐voltage electrolysis, thus producing value‐added byproducts, such as glucaric acid^[^ ^17^ ^]^ from glucose and furandicarboxylic acid^[^ ^18^ ^]^ from hydroxymethylfurfural. However, these chemicals are often expensive and derived from edible biomass, conflicting with the global food supply. In this context, lignin is an ideal alternative because it has no such issues, characterized by a massive annual production of ≈50 million tons as by‐products.^[^ ^19^ ^]^ Recently, we and other researchers reported that redox‐active homogeneous catalysts, such as Fe^3+^ and phosphomolybdic acid (PMA), can extract and store electrons and protons upon lignin oxidation while producing aromatic chemicals as byproducts.^[^ ^20^, ^21^ ^]^ The stored electrons and protons can be extracted on demand from the catalysts at a potential of <1.0 V versus RHE. Despite such promising results, these approaches are still in their infancy, with many challenging issues: discontinuous and time‐consuming multi‐step batch reactions, value‐added byproduct separation, and a gradual decrease in the hydrogen production rate owing to the consumption of the stored electrons/protons. Studies utilizing any type of biomass and its derivatives can also suffer from similar issues;^[^ ^22^ ^]^ however, these studies have been limited to proof‐of‐concept demonstrations without properly addressing these issues.

**Scheme 1:**
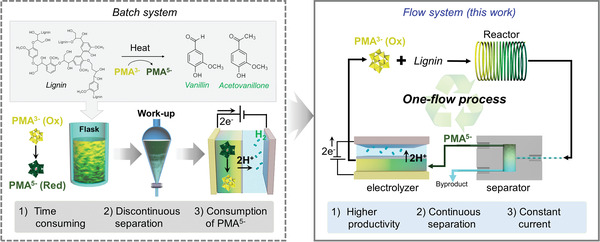
Graphical illustration explaining the motivation of this study on continuous lignin valorization and low‐voltage hydrogen production based on modular flow reactors. Continuous‐flow reactors, in‐line separators, and flow electrolyzer modules are integrated for one‐flow processing.

In this study, we developed a modular flow system for continuous‐flow electrochemical lignin valorization and low‐voltage hydrogen production to address these issues (**Scheme** **1**). Briefly, we developed fluidic functional modules for lignin depolymerization with the reduction of PMA^3−^ to PMA^5−^, byproduct separation, and electrolysis with the reactivation of PMA^5−^ to PMA^3−^. The integration of these modules enabled effective lignin depolymerization, real‐time separation and enrichment of aromatic byproducts, and more energy‐efficient hydrogen production. Particularly, the unique advantages of fluidic systems, such as effective mass and heat transfer, significantly reduced the reaction time for the PMA reduction via lignin depolymerization from 12 h to 32 min while improving the mass productivity of valuable byproducts, such as vanillin and acetovanillone (0.5 and 0.17 mg h^−1^, respectively). Hydrogen was stably produced at a current density of 20.5 mA cm^−2^ at 1.5 V without performance degradation via real‐time PMA^3−^ recycling. This study can provide insights into the design of novel electrochemical systems coupled with biomass utilization, thus establishing a foundation for the realization of green and economic hydrogen production.

## 2 Results and Discussion

We designed the overall continuous cycle for lignin depolymerization and low‐voltage hydrogen production based on the identification of modularizable elementary processes. We selected PMA, with a molecular formula of H_3_PMo_12_O_40_, as a homogeneous catalyst for oxidative lignin depolymerization and an electron and proton mediator owing to their high acidity and reversible redox behavior at moderate potentials.^[^ ^23^ ^]^ Additionally, the catalyst exhibited a unique color change from yellow to dark green upon reduction, allowing facile monitoring of the overall process.

**Scheme 2:**
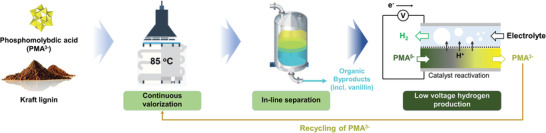
Illustration of modular flow reactors for valorization of kraft lignin and low‐voltage hydrogen production.

Based on our previous studies,^[^ ^21^ ^]^ we identified four functional units: 1) injection, mixing, and heating of aqueous solutions of lignin and PMA for oxidative lignin depolymerization; 2) extraction of aromatic byproducts using chloroform; 3) separation of the aqueous and organic phases; and 4) low‐voltage hydrogen production using the reduced PMA, followed by oxidized PMA recycling for lignin depolymerization. Each module was designed, optimized, and then integrated into the overall continuous‐flow system. We hypothesized that flow reactors can significantly enhance and accelerate the overall process via a continuous oxidized PMA (PMA^3−^) supply for lignin valorization and the reduced PMA (PMA^5−^) for low‐voltage hydrogen production. PMA becomes deactivated for lignin oxidation once it is reduced from PMA^3−^ to PMA^5−^; the depletion of the reduced PMA during electrolysis decreases the hydrogen production rate (**Scheme** **2**). However, the fluidic systems can further facilitate the overall process owing to their unique advantages for effective mass and heat transfer.^[^ ^24^, ^25^, ^26^, ^27^ ^]^

### 2.1 Continuous Depolymerization of Lignin

For continuous biomass oxidation, we designed a flow reaction platform (FRP) based on our previous reports.^[^ ^25^ ^]^ The FRP was compactly fabricated via thermocompression bonding of Teflon plates with flow channels (width: 1 mm; height: 1 mm) (Figure S1, Supporting Information). Three FRPs, each having a channel volume of 4 mL, were integrated with a heat exchanger module and connected in series. The aqueous solutions of lignin in 1.0 m H_2_SO_4_ and PMA were injected into the FRP through a static mixer for efficient mixing. The FRP allowed for safe operation with no leaking and corrosion even under harsh conditions owing to its chemical and mechanical robustness (tolerant at a pH range of 0–14 and up to 500 psi).

**Figure 1:**
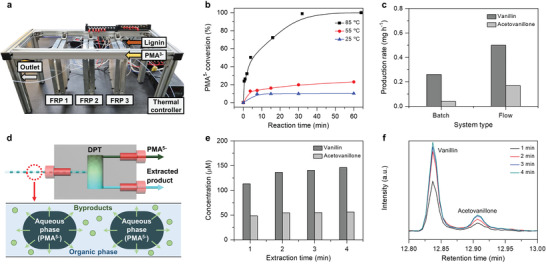
Continuous biomass valorization and separation of value‐added chemicals. Continuous‐flow systems for a–c) the depolymerization/valorization of lignin and d,e) separation of the lignin‐derived aromatic compounds. a) Photograph of the Flow Reaction Platform (FRP) with heat exchange for lignin depolymerization. b) Effect of reaction time and temperature on the reduction of PMA by lignin. c) Comparison of the production rates of lignin‐derived value‐added chemicals in the batch and flow systems. d) Working principles of in‐line extraction and density‐based phase separation tank (DPT) modules. e) Effect of the retention time on the extraction efficiency of vanillin and acetovanillone from the aqueous 1 m H_2_SO_4_ solution using chloroform, as investigated by f) GC‐MS analysis.

Using the FRP for continuous‐flow reactions, we examined the effect of the flow rate (i.e., residence time) on the efficiencies of lignin depolymerization and electron/proton extraction (**Figure** **1**a). We dispersed kraft lignin in 1 m H_2_SO_4_ at 0.092 g mL^−1^, where no residual particulate lignin was observed (Figure S2, Supporting Information), and PMA at 0.5 m in 1 m H_2_SO_4_ (Figure 1b). Upon mixing of lignin and PMA, the following redox reactions were expected according to recent studies^[^ ^21^, ^28^ ^]^

(1) $$\text{Overall:}\;\text{Lignin}+{\mathrm{PMA}}^{3-}\to {\mathrm{Lignin}}_{\mathrm{ox}}+{\mathrm{PMA}}^{5-}$$

(2) $$\text{Oxidation:}\;\text{Lignin}\to {\mathrm{Lignin}}_{\mathrm{ox}}+2H^{+}+2e^{-}$$

(3) $${\text{Reduction:}\;\text{PMA}}^{3-}+2H^{+}+2e^{-}\to {\mathrm{PMA}}^{5-}$$

The degree of PMA reduction was monitored using an UV–vis spectrophotometer (Figures S3 and S4, Supporting Information). The reduction degree was highly dependent on the temperature and became rapidly saturated, regardless of the temperature. PMA was completely reduced within 32 min (i.e., 0.375 mL min^−1^) of the residence time at 85 °C, whereas only a small portion of PMA was reduced at low temperatures (10% at 25 °C and 23% at 55 °C). Compared with the batch counterpart, the continuous‐flow system allowed for a more efficient/rapid depolymerization of lignin and extraction of electrons and protons. For example, the former resulted in only ≈20% PMA reduction under the same conditions (Figure S5, Supporting Information). Moreover, electrons and protons were extracted at 1.09 and 5.43 mmol g^−1^ for the batch and continuous‐flow systems, respectively. Additionally, our flow system substantially enhanced the production rate of the value‐added chemicals, such as vanillin and acetovanillone, upon lignin depolymerization. The lignin depolymerization degree was significantly improved at higher temperatures (Figures S6 and S7, Supporting Information). During the complete reduction of PMA for 32 min at 85 °C, 20.6% of lignin was decomposed. At a higher temperature, the conversion yield of vanillin and acetovanillone decreased although the absolute amount of the produced compounds increased. The decreased conversion yield at a higher temperature can be attributed to severe decomposition of lignin to smaller molecules^[^ ^29^ ^]^ and/or recoupling of intermediate radical compounds.^[^ ^30^ ^]^ Flow (or batch) systems produced vanillin at 0.5 mg h^−1^ (0.25 mg h^−1^) and acetovanillone at 0.17 mg h^−1^ (0.04 mg h^−1^) from 1 g of lignin (Figure 1c). These enhancements were attributed to the unique feature of fluidic systems for efficient mass and heat transfer. We note that the production rate can substantially be improved further by scaling up the reactors. 2D ^13^C−^1^H heteronuclear single‐quantum correlation (HSQC) nuclear magnetic resonance (NMR) analysis (**Table** **1** and Figure S8, Supporting Information) revealed that the formation of vanillin and acetovanillone resulted from the selective cleavage of *β*‐O‐4 links in lignin.

**Table 1:** Relative contents of the major links with respect to the total aromatic units in lignin before and after oxidation with PMAs using the continuous‐flow reactor. Contents were calculated based on 2D‐NMR analysis

Major links	Content [%]	
	Before	After
*β*‐O‐4	9.3	4.32
*β*‐5	1.59	1.45
*β*‐*β*	1.13	0.71

### 2.2 Continuous‐Flow Separation of Aromatic Byproducts and Reduced PMA

Next, we developed in‐line modules for the selective extraction of valuable aromatic byproducts from the aqueous reaction mixture. We selected chloroform as an extracting solvent because it can selectively recover aromatic byproducts, but cannot extract strongly ionic and polar PMA^5−^ from the aqueous phase. For in‐line separation, we used a commercially available T‐mixer to generate a segmented flow, where each aqueous slug was sandwiched between thin layers of the continuous organic phase for efficient extraction (Figure 1d). Such a slug flow can not only increase the interfacial area between the two phases, but it also induces an internal vortex via the sheer force between them^[^ ^31^, ^32^ ^]^ (Figure S9, Supporting Information). Consequently, such a continuous‐flow system can significantly enhance the mass transfer and extraction efficiency, as compared with the batch system.^[^ ^31^, ^32^ ^]^ At a constant chloroform flow rate of 0.375 mL min^−1^, which was optimized for lignin depolymerization, the extraction time was controlled by varying the length of the capillary tubing (I.D.: 1 mm): 96, 191, 287, and 382 cm for extraction times of 1 to 4 min. We note that the recirculating vortex effect could be reduced if the distance between the slugs was different from the slug length (i.e., the chloroform flow rate was different from that of the aqueous solution). According to the gas chromatography‐mass spectrometry (GC‐MS) analysis, vanillin and acetovanillone were produced at 146 × 10^−6^ and 56 × 10^−6^ m, respectively (Figure 1e,f), with complete extraction within 3 min (Figures S10–S12, Supporting Information). After extraction, only reduced PMA was identified with no detectable lignin depolymerization byproducts in the aqueous solution (Figure S12, Supporting Information). After extraction using a segmented flow, we separated the aqueous solution, containing the reduced PMA, and chloroform, containing the byproducts, using a density‐based phase‐separation tank (DPT) (Figure 1d). The segmented flow of the aqueous solution and chloroform was injected into a single inlet in the middle of the DPT, phase‐separated owing to their density difference, and discharged through upper and lower outlets, respectively. For efficient separation, we designed the DPT based on computational fluid dynamics (CFD) simulations to suppress the formation of an emulsion between the aqueous solution and chloroform (Figure S13, Supporting Information). Consequently, we completely separated the aqueous solution and chloroform before electrolysis. The pre‐separation process was important for both hydrogen productions via electrolysis and byproduct recovery because the aromatic byproducts could be decomposed further to less valuable aliphatic molecules upon electrolysis and cause electrode fouling.^[^ ^33^, ^34^, ^35^ ^]^ Moreover, continuous operation of the separation modules minimized consumption of chloroform by obtaining the enriched vanillin up to 121 × 10^−3^ m (Figure S14, Supporting Information), which was significantly concentrated over a sub‐mM scale in the batch process. Notably, the recycling of volatile chloroform would be feasible by conventional post‐separation for a greener process.

### 2.3 Low‐Voltage Hydrogen Production Using Reduced PMA

**Figure 2:**
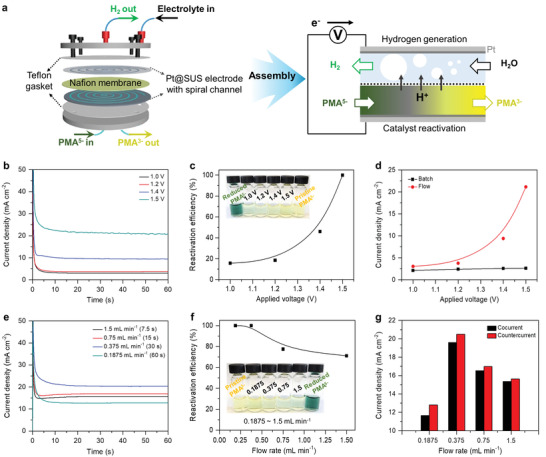
Continuous hydrogen production and catalyst reactivation. a) Flow scheme and optimization of PMA^5−^ reactivation and the green hydrogen production process in a continuous‐flow electrolyzer. Effect of various applied voltages (flow rate: 0.375 mL min^−1^; *t* ^R^: 30 s) to the b) current density and c) reactivation efficiency of PMA^5−^. d) Current density profile at various applied voltages dependent on the different systems. Effect of various applied flow rates (voltage: 1.5 V) to the e) current density profiles and f) reactivation efficiency of PMA^5−^. g) Current density profile at various applied flow rates dependent on the flow direction.

After separation, the reduced PMA in the aqueous 1.0 m H_2_SO_4_ solution was re‐oxidized in an electrolyzer for low‐voltage hydrogen production and recycled for repeated use during lignin depolymerization. **Figure** **2**a shows the structure of a continuous‐flow electrolyzer that is composed of two electrode chambers separated with the Nafion membrane. Briefly, corrosion‐resistant stainless steel 304 (SUS304) plates were engraved with a fluid channel^[^ ^36^ ^]^ (width: 0.8 mm; height: 300 µm; length: 62.5 cm; and channel surface area: 5.08 cm^2^). The length of the flow channel was determined based on the flow rate for lignin depolymerization and byproduct separation (vide infra). The channel wall was coated with platinum via electron beam evaporation (Figure S15, Supporting Information). PMA^5−^ was only injected into the bottom channel (anode) and re‐oxidized to PMA^3−^ (i.e., reactivated for lignin depolymerization), supplying electrons and protons for the hydrogen evolution reaction (HER) on a cathode (Figure 2a).

Based on such settings, we investigated the effect of the applied voltage on the efficiencies of hydrogen production and PMA re‐oxidation (i.e., reactivation). We applied potentials of 1.0–1.5 V between the cathode and anode while maintaining a constant flow rate of 0.375 mL min^−1^ (retention time: 30 s) (Figure 2b,c, and Figure S16, Supporting Information). The applied potential significantly influenced the rate of hydrogen production and the efficiency of PMA reactivation. At 1.0 and 1.2 V, hydrogen was produced at a current density of 3.7 mA cm^−2^ with a PMA reactivation efficiency of ≈18%. When increasing the applied voltage to 1.4 and 1.5 V, we observed current densities of 10 and 20.5 mA cm^−2^ with reactivation efficiencies of 46% and 100%, respectively. No or negligible current flow was measured when injecting PMA^3−^ or only unreacted lignin, confirming the role of PMA as an electron and proton mediator (Figure S17, Supporting Information). We note that the inherently small dimension of continuous‐flow devices is also beneficial for ensuring a low ionic resistance^[^ ^37^, ^38^, ^39^ ^]^ (i.e., ohmic drop) and improving the efficiency of electrochemical hydrogen production compared with the bulk counterpart, as evident from Figure 2d.

Next, we examined the influence of the flow rate and flow directions of the cathode and anode electrolytes on the efficiency of our flow electrolyzer. The flow rate (or retention time, *t* ^R^) of the PMA^5−^ solution was varied from 0.1875 (*t* ^R^ = 1 min) to 1.5 mL min^−1^ (*t* ^R^ = 7.5 s) at a constant applied potential of 1.5 V. The retention time was proportional to the reactivation efficiency, but inversely proportional to the current density. Expectedly, the optimum flow rate was 0.375 mL min^−1^ (*t* ^R^ = 30 s), which achieved both the complete reactivation of the reduced PMA (Figure 2e,f) and the maximum current density (Figure 2g). We then investigated the effect of the relative direction of the electrolyte flows in the cathode and anode compartment at a constant potential of 1.5 V. We note that concurrent (or countercurrent) flow implies parallel (or antiparallel) electrolyte flows in the anode and cathode channels. The countercurrent cases always showed higher current densities than the concurrent cases because the former could generate a higher concentration gradient and thus faster proton diffusion across the electrolyte solution between the cathode and anode than the latter.^[^ ^40^ ^]^ However, the difference between their current density gradually decreased at higher applied voltages owing to the smaller difference in the concentration gradient (Figure 2g). Our flow electrolyzer showed a near‐unity faradaic efficiency (Figure S18, Supporting Information). In contrast, an electrolyzer without a continuous supply of fresh PMA^5−^ showed a rapid decline in the hydrogen production rate owing to the consumption and depletion of the reduced PMA^5−^ (Figure S19, Supporting Information).

### 2.4 Continuous Valorization of Lignin and Low‐Voltage Hydrogen Production

**Figure 3:**
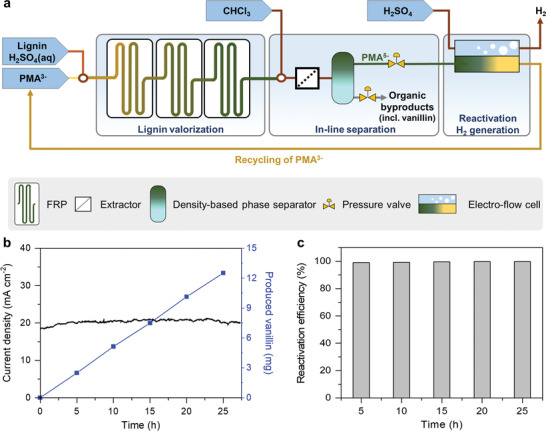
Integrated sustainable continuous‐flow process for lignin valorization, hydrogen generation, and catalyst reactivation. a) Flow scheme of the overall process. b) Current density profiles of a reduced PMA solution and the amount of produced vanillin measured every 5 h. c) Reactivation efficiency of PMA^5−^ during a long‐term process operation.

Lastly, we integrated all of the functional modules (i.e., FRPs, extractor, DPT, and electrolyzer) for continuous lignin valorization, value‐added byproduct separation, and low‐voltage hydrogen production (Figure S20, Supporting Information). Feedstock solutions of lignin, PMA^3−^, chloroform, and electrolyte were individually stored and injected continuously at 0.375 mL min^−1^ using high performance isocratic pumps based on the optimization above. PMA was reduced in the FRPs, which was maintained at a constant temperature of 85 °C upon reaction with lignin, separated from the lignin byproducts, re‐oxidized in the electrolyzer, and recycled for repeated reaction with lignin (**Figure** **3**a). The integrated system was continuously operated without any noticeable performance degradation for at least 25 h. Hydrogen and vanillin were produced at constant rates of 20.5 mA cm^−2^ and 0.5 mg h^−1^, respectively (Figure 3b), via real‐time PMA^3−^ recycling without activity loss (Figure 3c). After 25 h, the integrated system produced a separated vanillin quantity of 12.5 mg. Our continuous‐flow system for continuous lignin valorization and hydrogen production has many advantages over its batch counterpart and conventional water electrolysis for hydrogen production (**Table** **2**). By replacing the OER with PMA‐mediated lignin oxidation, we substantially enhanced the rate and energy‐consumption of electrochemical hydrogen production. Our continuous‐flow system produced hydrogen at a current density of 20.5 mA cm^−2^ at 1.5 V, whereas the conventional water electrolysis can produce hydrogen at < 10 mA cm^−2^, even with expensive state‐of‐the‐art OER electrocatalysts (**Table** **3**).

**Table 2:** Comparison of hydrogen production methods dependent on different systems

	Water electrolysis	Biomass oxidation (Batch)	This work (Flow)
Half reactions	HER + OER	HER + Biomass oxidation	HER + Biomass oxidation
Oxidation catalyst	Precious metal‐based catalysts (e.g., Pt, Ir, Ru, Pd, etc.)	Earth‐abundant transition metal‐based molecular catalysts	Earth‐abundant transition metal‐based molecular catalysts
Current density at 1.5 V (Ref. [41, 42, 43, 44, 45, 46, 47, 48, 49])	≈10 mA cm^−2^	≈20 mA cm^−2 *^	20.5 mA cm^−2^
Current profile	Stable	Rapidly decreasing	Stable
Safety	High explosion risk due to O_2_	Low explosion risk (O_2_ free)	Low explosion risk (O_2_ free)
Additional value‐added product	None	Vanillin and acetovanillone	Vanillin and acetovanillone

*Note*: when using a batch‐type electrolyzer at 1.5 V, the current density was initially ≈20 mA cm^−2^, but rapidly converged to 2 mA cm^−2^ owing to reduced PMA^5−^ consumption.

**Table 3:** Comparison of the energy requirements for hydrogen production by utilizing electrocatalysts

Entry	System	Electrolyte	Product	Catalyst for oxidation half‐reactions	Current density [mA cm^−2^]	Applied voltage [V]
This work	Biomass oxidation	1.0 m H_2_SO_4_	H_2_ and Vanillin	PMA	20.5	1.5
Ref. [41]	Water splitting	1.0 m KOH	H_2_ and O_2_	Co(S_x_Se_1 −_ *_x_*)_2_	10	1.63
Ref. [42]	Water splitting	1.0 m KOH/ 0.5 m H_2_SO_4_	H_2_ and O_2_	Zn_0.1_Co_0.9_Se_2_	10	1.6
Ref. [43]	Water splitting	1.0 m KOH	H_2_ and O_2_	(Ni,Co)_0.85_Se	10	1.65
Ref. [44]	Water splitting	1.0 m KOH	H_2_ and O_2_	Se(NiCo)S_x_/(OH)*_x_*	10	1.6
Ref. [45]	Water splitting	0.05 m H_2_SO_4_	H_2_ and O_2_	RuIr‐NC	10	1.49
Ref. [46]	Water splitting	1.0 m KOH	H_2_ and O_2_	W_2_N/WC	10	1.58
Ref. [47]	Water splitting	1.0 m NaOH	H_2_ and O_2_	NiMoO_4‐_ *_x_*/MoO_2_	10	1.56
Ref. [48]	Water splitting	1.0 m KOH	H_2_ and O_2_	Porous MoO_2_	10	1.53
Ref. [49]	Water splitting	1.0 m KOH	H_2_ and O_2_	VOOH nanosphere	10	1.62

The performance could be improved further via the exploration of optimal lignin, the development/utilization of redox‐active catalysts, finely optimizing the electrolysis conditions, and scaling up of the reported systems for practical applications. In addition to hydrogen, value‐added chemicals, such as vanillin and acetovanillone, were produced as lignin depolymerization byproducts. Moreover, we could also minimize or avoid safety concerns caused by the mixing of hydrogen and oxygen in conventional water electrolysis. Although we here utilized kraft lignin which account for about 85% of the overall lignin production, one can also utilize various types of more reactive lignin,^[^ ^50^, ^51^, ^52^ ^]^ such as alkali lignin, lignosulfonate, swollen residual enzyme lignin, double enzyme lignin, organosolv lignin, etc., that have a higher *β*‐O‐4 linkage and G contents than kraft lignin to improve both the lignin depolymerization/valorization activity and the hydrogen production efficiency. It is reported that the contents of *β*‐O‐4 linkage and G units play a crucial role on the efficiency and selectivity of lignin depolymerization (Figure S21, Supporting Information).^[^ ^50^ ^]^ The efficiency of the current system can be also improved by increasing the solubility of lignin and/or developing solid/liquid reactors because they allow the utilization of more concentrated PMA for rapid lignin valorization and efficient hydrogen production.

## 3 Conclusion

In summary, for the first time, we reported the development of modular flow reactors for continuous‐flow lignin‐valorization and low‐voltage hydrogen production. We designed and fabricated the functional modules for 1) biomass depolymerization to generate value‐added byproducts, electrons, and protons using a redox‐active catalyst PMA; 2) extraction of the byproducts using organic solvents (e.g., chloroform); 3) separation of the organic solvent and extracts; and 4) low‐voltage hydrogen production upon reactivation (re‐oxidation) of the reduced catalysts. Each module was tested to explore the optimal design and operation conditions. Consequently, the integrated modular system continuously produced hydrogen and value‐added byproducts of lignin depolymerization (i.e., vanillin and acetovanillone) more efficiently than its batch and bulk counterparts owing to efficient real‐time catalyst recycling and byproduct separation. Our results can provide insights into the design and realization of continuous‐flow reactors as end‐to‐end solution for biomass utilization and more economically viable electrochemical hydrogen production systems.

## Conflict of Interest

The authors declare no conflict of interest.

## Supporting information

Supporting Information

## Data Availability

The data that support the findings of this study are available from the corresponding author upon reasonable request.
