# Supplementary material for: Modular Flow Reactors for Valorization of Kraft Lignin and Low‐Voltage Hydrogen Production
Source: Adv Sci (Weinh). 2022 Oct 26;9(35):2204170. doi: 10.1002/advs.202204170 (PMC9762309; doi:10.1002/advs.202204170)
Supplement: Supplementary file 1 — Supporting Information [file ADVS-9-2204170-s001.pdf]

ADVANCED SCIENCE
Open Access

Supporting Information

for *Adv. Sci.*, DOI 10.1002/advs.202204170

Modular Flow Reactors for Valorization of Kraft Lignin and Low-Voltage Hydrogen Production

Se-Jun Yim, Hyeonmyeong Oh, Yuri Choi, Gwang-Noh Ahn, Chae-Hyeon Park, Yong Hwan Kim, Jungki Ryu and Dong-Pyo Kim**

Supporting Information

Modular Flow Reactors for Valorization of Kraft Lignin and Low-Voltage Hydrogen Production

Se-Jun Yim†, Hyeonmyeong Oh†, Yuri Choi, Gwang-Noh Ahn, Chae-Hyeon Park, Yong Hwan Kim, Jungki Ryu and Dong-Pyo Kim**

S. Yim, G. Ahn, C. Park, Prof. D. Kim
Department of Chemical Engineering
Pohang University of Science and Technology (POSTECH)
Pohang 37673, Republic of Korea
E-mail: dpkim@postech.ac.kr (D.K.)

H. Oh, Y. Choi, Prof. Y. Kim, Prof. J. Ryu
Department of Energy Engineering
School of Energy and Chemical Engineering
Ulsan National Institute of Science and Technology (UNIST)
Ulsan 44919, Republic of Korea

H. Oh, Y. Choi, Prof. J. Ryu
Emergent Hydrogen Technology R&D Center
Ulsan National Institute of Science and Technology (UNIST)
Ulsan 44919, Republic of Korea

Prof. J. Ryu
Graduate School of Carbon Neutrality
Ulsan National Institute of Science and Technology (UNIST)
Ulsan 44919, Republic of Korea
E-mail: jryu@unist.ac.kr (J.R.)

† These authors contributed equally to this work.

Table of Contents

1. Experimental section···S1

2. Flow reaction platforms (FRPs) for continuous lignin depolymerization······S3

2.1 FRP design ······S3

2.2 Preparation of lignin solution······S3

2.3 Calibration curve for monitoring lignin depolymerization and PMA reduction······S4

2.4 Chemical structure of lignin before and after depolymerization by PMA······S7

3. Extraction and separation of lignin depolymerization byproducts ······S8

3.1 Extraction of lignin byproducts ······S8

3.2 Density-based phase-separation tank (DPT) for separating aqueous and organic phases ······S10

4. Flow electrolyzers for low-voltage hydrogen production and PMA reactivation······S12

4.1 Fabrication of microchannel flow electrolyzer ······S12

4.2 Optimization of microchannel flow electrolyzer ······S12

4.3 Flow vs. batch electrolyzer ······S14

5. Integrated flow systems ······S14

6. Potential effect of types of lignin on the lignin valorization ······S15

7. References ······S16

Experimental

Materials

Phosphomolybdic acid ($H_3[PMo_{12}O_{40}]$, PMA), kraft lignin, chloroform, and sulfuric acid were purchased from Sigma-Aldrich (USA). SUS304 plates were purchased from SKB TECH (South Korea). Nafion^{TM} 117 was purchased from the Fuel Cell Store (USA). T-junctions, flangeless fittings, and

polytetrafluoroethylene (PTFE, 1 mm i.d. 1mm) tubes were purchased from IDEX Health & Science (WA, USA). Syringe pumps (Harvard apparatus, PHD 22/2000 Hpsi, PHD Ultra) and high performance isocratic pumps (SP-930D, Younglin, Korea) were used.

Preparation of PMA and lignin solution

PMA was dissolved to 0.5 M in 1 M H_2SO_4. After sonication for 2 h, a homogeneous yellow colored solution was obtained. The lignin solution was prepared by dissolving 9.2 g of lignin in 100 mL of 1 M H_2SO_4 (100 mL) and then by doubly filtering it via centrifugation at 4,000 RPM for 10 min and vacuum filtered to avoid microchannel blocking.

Fabrication of functional modules for continuous-flow reactors

Details on the design and optimization of each module are described in the manuscript and ESI.

Characterization

The concentrations of the reduced PMAs were confirmed by measuring the absorbance spectra with a V-730 UV−visible spectrophotometer (JASCO, Japan). The lignin structure was analyzed with a VNMRS 600 nuclear magnetic resonance (NMR) spectrometer (Agilent, USA).

Electrochemical analysis

Chronoamperometry (CA) was measured using a SP-150 Biologic potentiostat (BioLogic Science Instruments, France). Samples for GC analyses were collected from the outline of the tube from the cathode component and analyzed with a GC-2010 Plus gas chromatograph (Shimadzu Co., Japan).

Quantification of vanillin and acetovanillone

After the continuous separation process to extract vanillin and acetovanillone, 1 μL of n-decane was added to 2 mL of a chloroform solution as an internal standard for the GC-MS analysis. To set the calibration curve for vanillin and acetovanillone to verify the product yield, the intensity, according to the concentrations of the vanillin (**Figure S10b**) and acetovanillone (**Figure S10c**) solutions, was measured. The GC-MS spectra were measured with a 450-GC gas chromatograph and a 320-MS mass spectrometer

(Bruker, USA), equipped with an Rtx-5MS S5 capillary column (30 m × 0.25 mm × 0.25 mm; Restek). Split injections (1 μL) were performed with a GC Pal autosampler (CTC Analytics AG, Switzerland) at a split ratio of 25:1 using helium as a carrier gas.

Computational fluid dynamics simulation setup

The fluid flow inside a reactor and separator can be described by the incompressible Navier-stokes equation, together with a mass-conservation equation. Assuming steady state, the governing equation for fluid flow can be simplified to the following:

$$\rho\, \mathrm{v} \cdot \nabla \mathrm{v} = -\nabla \mathrm{p} + \mu \nabla^2 v + \rho g \text{ (Navier} - \text{Stokes equation) and} \quad (1)$$

$$\nabla \cdot \mathrm{v} = 0 \text{ (Mass conservation equation),} \quad (2)$$

where ρ [kg m^{-3}] is the fluid density, v [m s^{-1}] is the fluid linear velocity, p [Pa] is the pressure, μ [kg m^{-1} s^{-1}] is the fluid dynamic viscosity and g (= 9.8m s^{-2}) is the gravitational acceleration. The governing equations were solved with appropriate boundary conditions (i.e., no slip boundary conditions on the channel walls, mass flow rate for the inlets, outflow conditions for the outlets, and zero normal gradients for all of the flow variables, except for the pressure). The equations were discretized using the finite-volume method using the commercial numerical software The FLUENT 2020 R1 (ANSYS, INC.). The physical properties (density and viscosity) of water (998 kg m^{-3} and 0.001793 kg m^{-1} s^{-1}, respectively) and chloroform (1,490 kg m^{-3} and 0.00053 kg m^{-1} s^{-1}, respectively) at 25 ^{o}C as a carrier solvent were used for the calculation of fluid properties.

2. Flow reaction platforms (FRPs) for continuous lignin depolymerization

2.1 FRP design

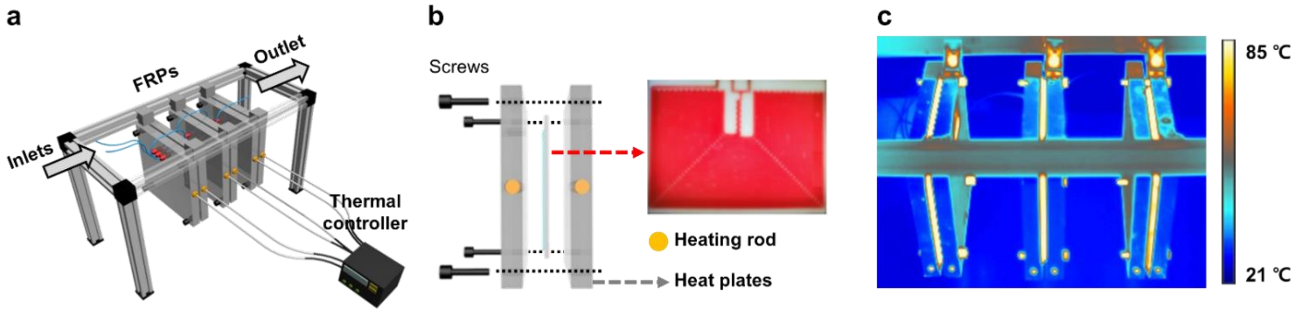

Figure S1. FRPs for continuous-flow oxidative lignin depolymerization. (a) Schematic illustration of the FRP system equipped with a heating module. (b) Photograph of FRP 1 filled with red dye (right) and a cross-sectional diagram of the flow reactor sandwiched between two metal frames, which incorporate rod-shaped heaters (left). (c) Temperature profile of the FRPs measured by an IR camera.

2.2 Preparation of lignin solution

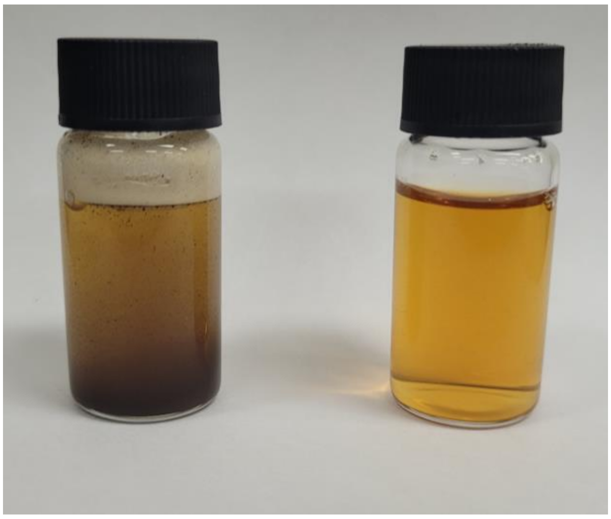

Figure S2. Photographs of solutions with different amount of kraft lignin. (Left) The addition of excess lignin led to aggregation and precipitation. Sonication, centrifugation, and filtration of the left solution results in homogenous solution of lignin, of which concentration is 0.092 g mL^{-1}. Based on this finding, we determined the solubility limit of kraft lignin in 1 M H_2SO_4 to be 0.092 g mL^{-1}.

2.3 Calibration curve for monitoring lignin depolymerization and PMA reduction

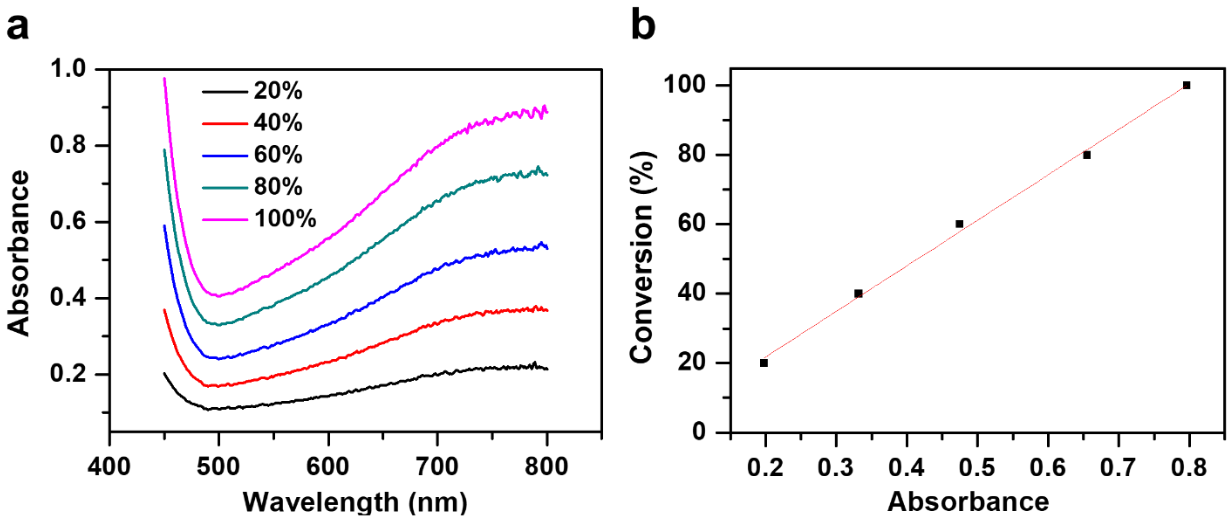

Figure S3. (a) Correlation between the concentration and absorbance of the reduced PMA spectra of the PMA solutions with different degrees of reduction from PMA^{3-} to PMA^{5-}. (b) Relationship between the degree of PMA reduction and absorbance at 700 nm (conversion was calculated by following the Beer-Lambert law). Samples (20 μL) were collected at steady-state, diluted 250 times with deionized water, and analyzed via a UV-vis spectrophotometer.

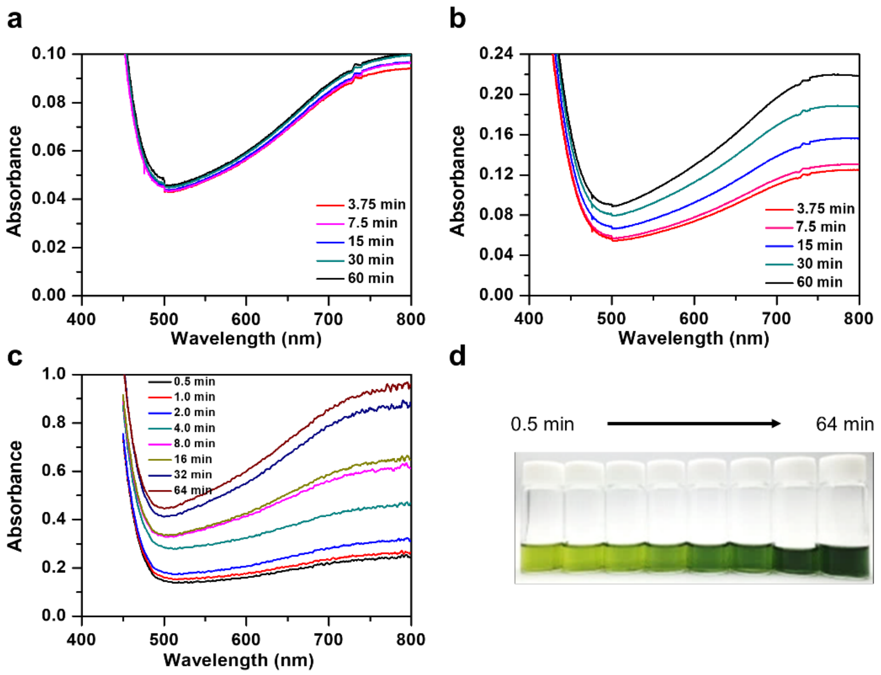

Figure S4. Effect of temperature and retention time on the reduction of PMA by lignin in FRP-based microfluidic reactors. (a–c) UV-vis absorbance spectra and (d) a photograph of the solution mixtures of PMA and lignin, which were incubated at (a) 25 °C, (b) 55 °C, and (c, d) 85 °C for various retention times (concentrations of PMA^{3-} and lignin were 0.5 M and 0.092 g mL^{-1}, respectively, in 1.0 M H_2SO_4).

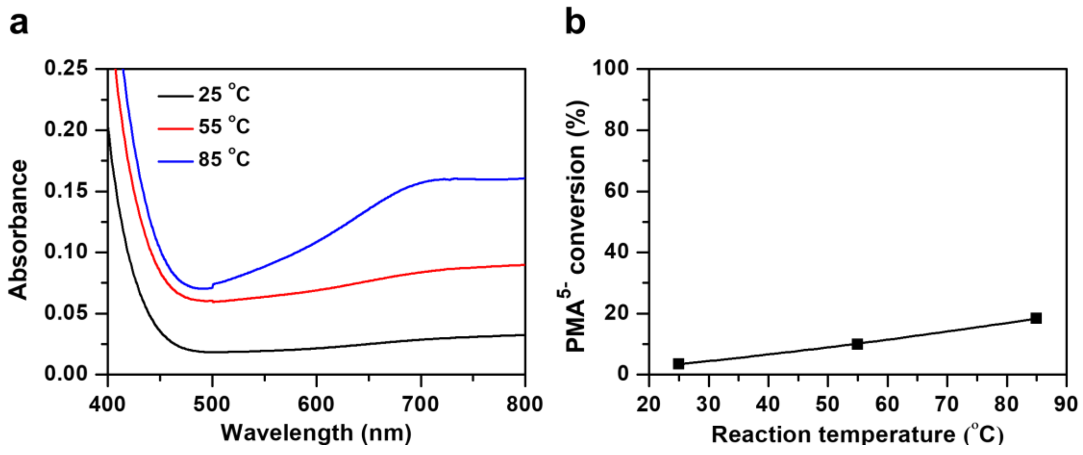

Figure S5. Effect of temperature on the reduction of PMA by lignin for 32 min in batch reactors. (a) UV-vis absorbance spectra of the solution mixture of PMA and lignin incubated at different temperatures. (b) PMA reduction profile as a function of the reaction temperature. The concentrations of PMA^{3-} and lignin were 0.5 M and 0.092 g mL^{-1}, respectively, in 1.0 M H_2SO_4).

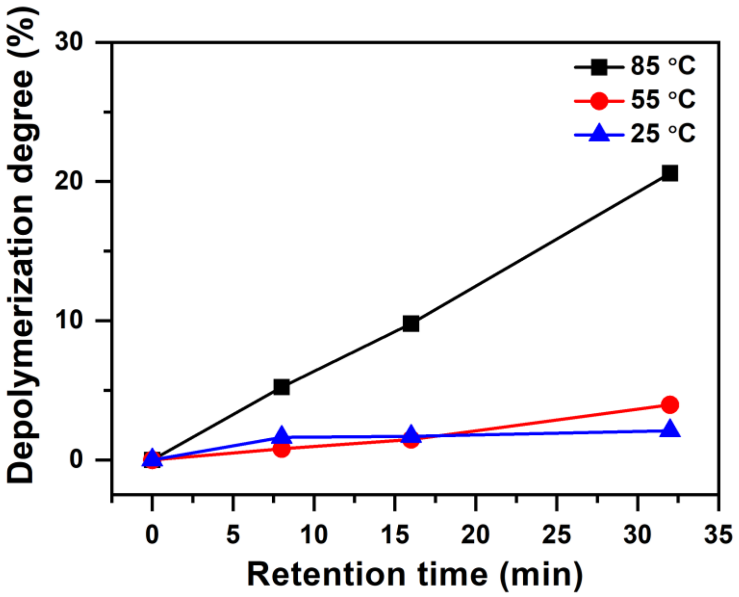

Figure S6. Effect of the reaction time and temperature on the depolymerization of lignin. The depolymerization degree was determined by comparing the mass of dissolved lignin before and after the reaction. Before the measurement, the dissolved PMA was removed by precipitation with the equivalent amount of CsCl. The depolymerization of lignin linearly increased with the incubation temperature and retention time. Due to the limited solubility and the complete reduction of PMA, however, the maximum retention time was limited to 32 min.

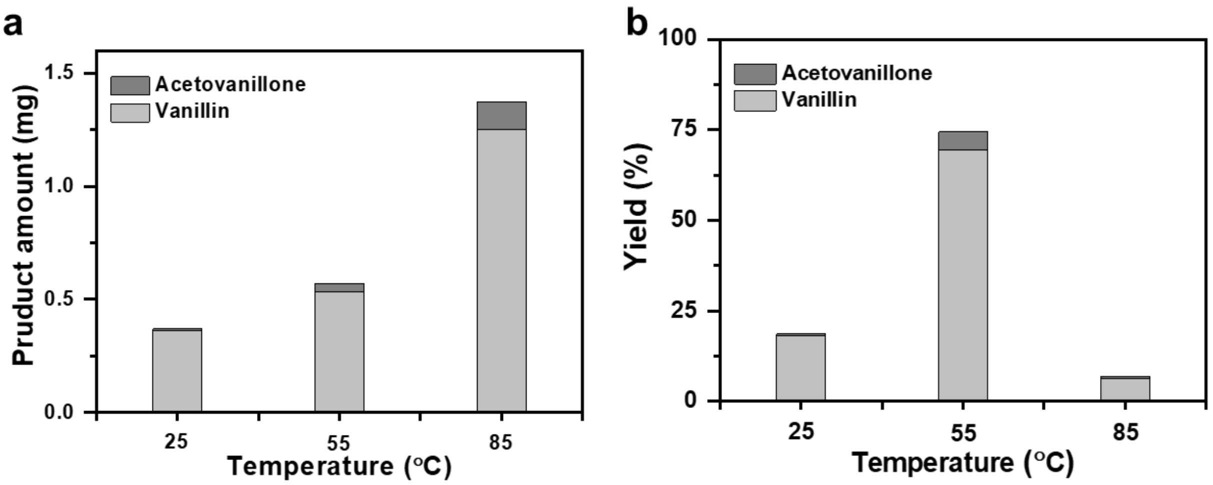

Figure S7. Effect of temperature on the production of the depolymerization byproduct of kraft lignin. (a) absolute amount of the produced vanillin and acetovanillone. (b) Conversion yield of the decomposed lignin to vanillin and acetovanillone. The conversion yield was calculated by dividing the weight of the produced vanillin and acetovanillone with the weight loss of lignin upon reaction, which is determined in Figure S5.

2.4 Chemical structure of lignin before and after depolymerization by PMA

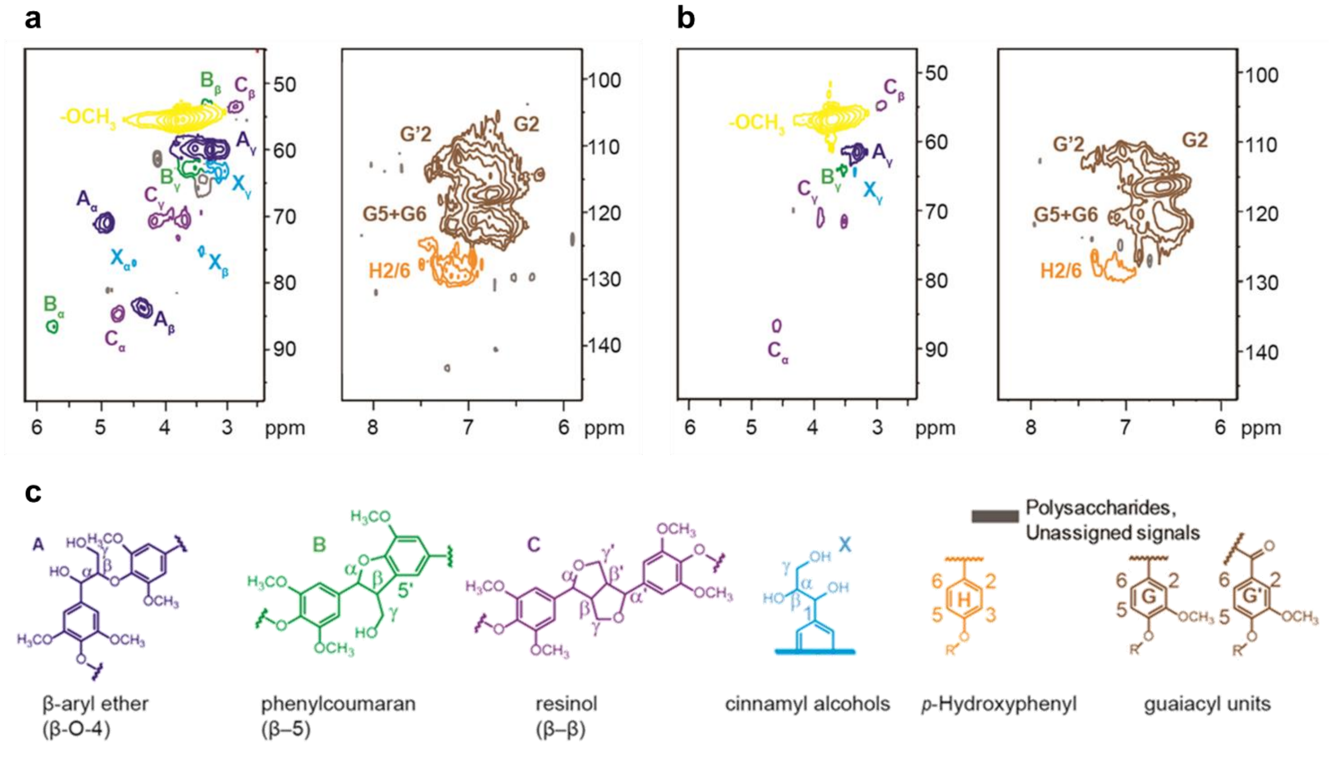

Figure S8. 2-D NMR analysis of lignin (a) before and (b) after the continuous-flow reaction with PMA for the identification and quantification of (c) various structural motifs of lignin. Lignin and PMA were reacted at 85 °C for 32 min using FRPs contours in the 2-D NMR spectra, color-coded according to the structural motifs in lignin. The quantification of G, S, and H units based on 2-D NMR analysis suggests that the kraft lignin tested is derived from softwood, as evidenced by the fact that it is mostly composed of G units (96.1%) with a small amount of H units (3.9%) and no detectable S units.

3. Extraction and separation of depolymerized lignin byproducts

3.1 Extraction of lignin byproducts

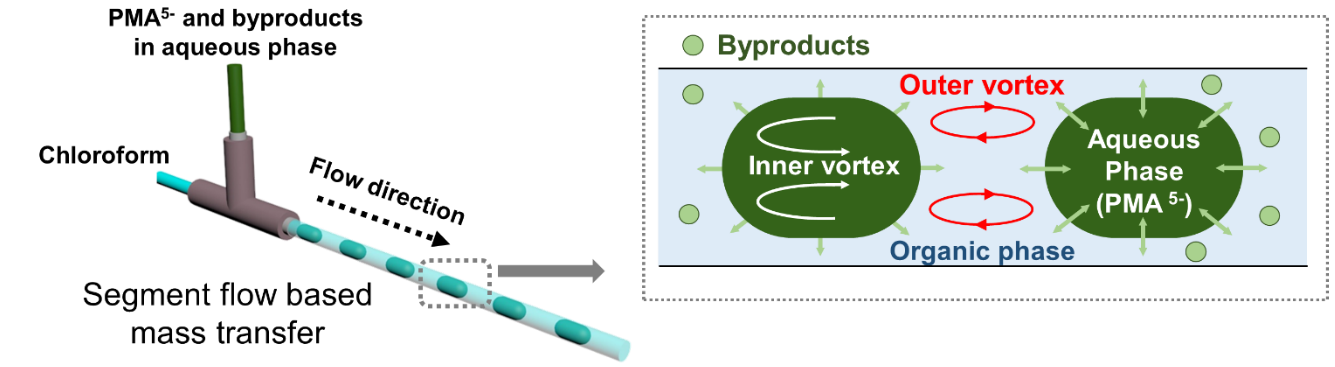

Figure S9. Generation of a segmented flow for the efficient extraction of lignin byproducts using a T-mixer. In the segmented flow, aqueous slugs were sandwiched between thin layers of the continuous organic phase, increasing the interfacial area between the two phases. Additionally, the shear force between the thin layer and slug flow induced an internal vortex in the top half of the slug and an opposing vortex flow in the bottom half of the slug. Simultaneously, the shear force between the channel wall and continuous phase adjacent to the slug induced two internal recirculating vortex flows. Consequently, the mass transfer was significantly enhanced, as compared with the batch counterpart[1,2].

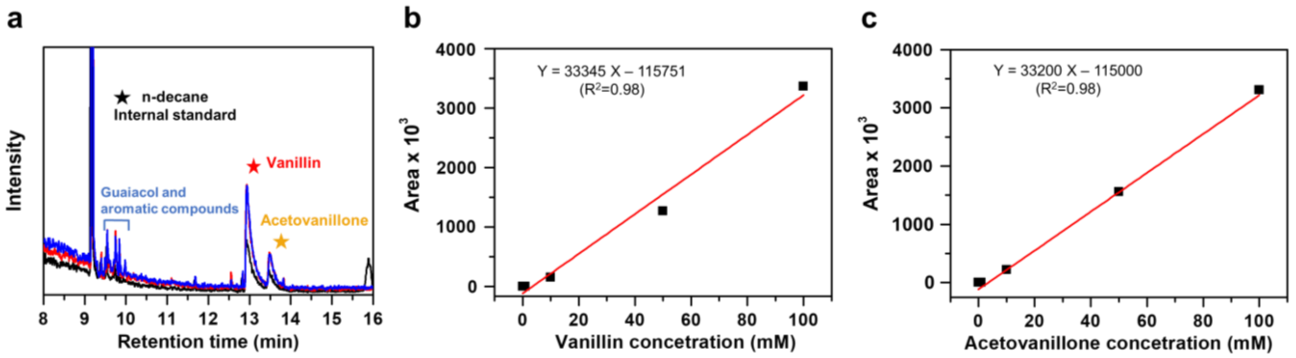

Figure S10. Calibration for estimating the extracted amount of vanillin and acetovanillone. (a) GC-MS spectra of the internal standard (n-decane) and lignin byproducts, such as vanillin and acetovanillone. Calibration curves of (b) vanillin and (c) acetovanillone. In addition to vanillin and acetovanillone, a small amount of guaiacol can be identified at 9.9 min. The rest of the peaks are thought to be related to various aromatic compounds.

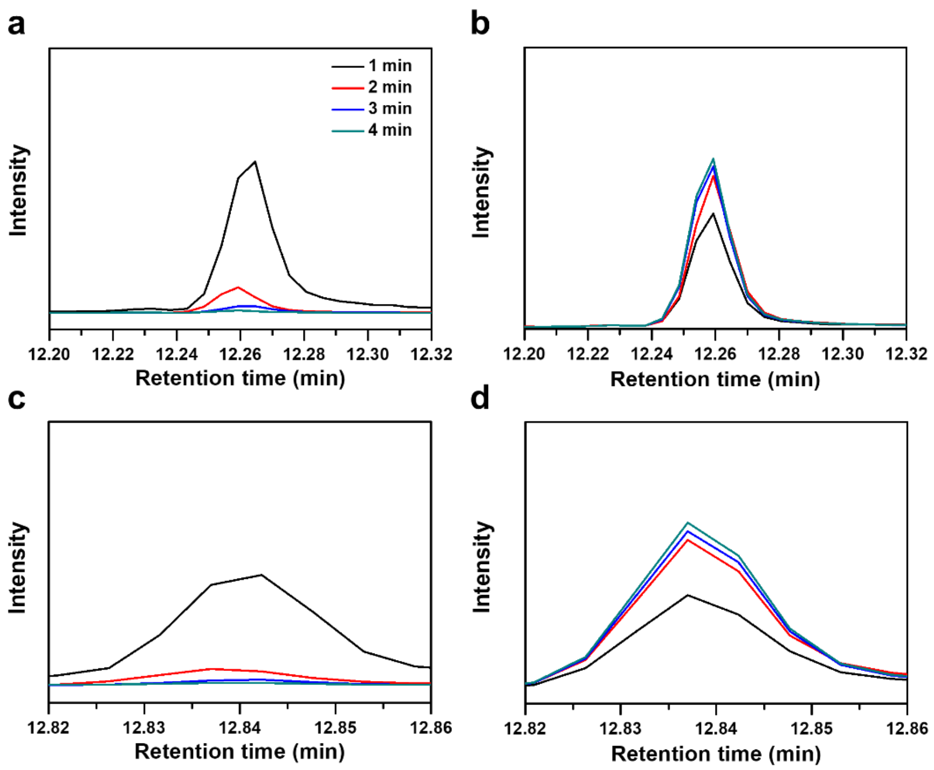

Figure S11. GC-MS analysis to investigate the effect of the extraction time on the extraction efficiency of the lignin byproducts using the method shown in Fig. S7. GC-MS spectra of (a, b) vanillin and (c, d) acetovanillone in (a, c) the aqueous solution and (b, d) chloroform after extraction for a given period.

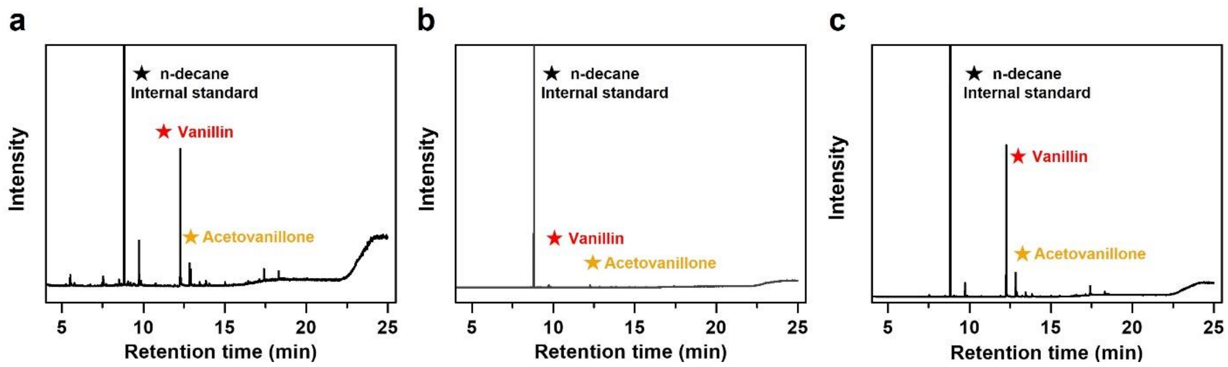

Figure S12. Distribution of PMA in the aqueous and organic phases after the continuous-flow extraction process. GC-MS spectra of (a) the crude mixture formed by the reaction between lignin and PMA before the extraction; (b) the aqueous and (c) organic phases after extraction. These results confirmed that PMA remained in the aqueous solution even while lignin byproducts were completely transferred to the organic phase.

3.2 Density-based phase-separation tank (DPT) for separating aqueous and organic phases

Chloroform, with a relatively higher density than water, was discharged to the outlet at the bottom. The pressure difference caused by the different heights of the outlets was controlled via the pressure control valve (Fig. S10a). For example, the organic and aqueous phases entering the DPT with the same flow rate ratio were discharged at the same quantity. Thus, by simply verifying the flow rate of the two outlets, we could modulate the liquid level in the DPT inner tank. However, only controlling the liquid level could not guarantee a 100 % separation efficiency because two immiscible phases can form an emulsion zone[3] that impedes effective separation. Therefore, to check the emulsion distribution inside the DPT and the actual separation performance, computational fluid dynamic (CFD) simulations and real experiments were performed to verify the separation efficiency of the DPT (Fig. S10b). Therefore, the emulsion zone formed with a thickness of 4.6 mm at the center height of the tank, indicating that the two liquid phases could be completely separated.

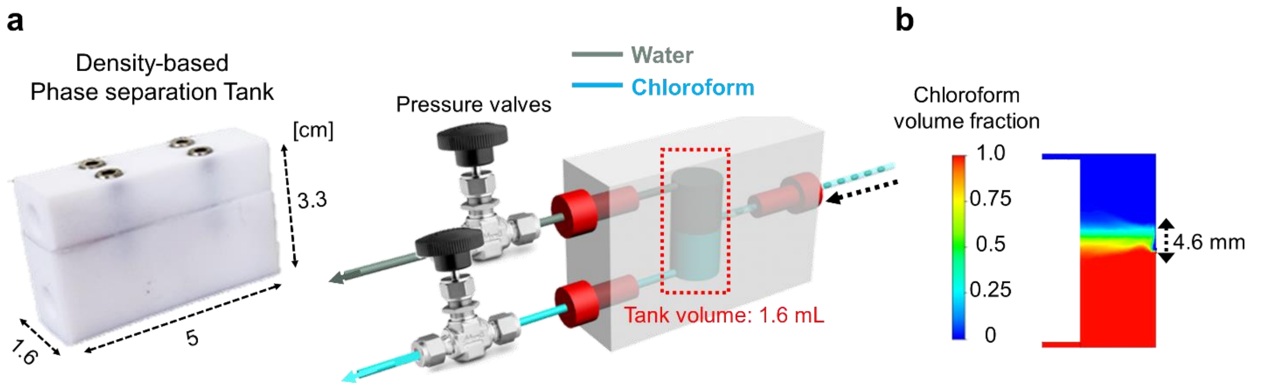

Figure S13. DPT for the separation of an immiscible liquid mixture, such as that of water and chloroform. (a) Actual image of the DPT connected with valve showing complete separation of water and chloroform. (b) Calculation of the water and chloroform volume fraction via computational fluid dynamic simulations (CFD).

We found the acceptable flow rate range for the complete separation of the aqueous solution and chloroform, as listed below.

Reaction solvent	Extraction solvent	Surface tension (mN/m)	Density at 25 °C	Acceptable Flow rate range	Separation efficiency	
					Simulation	Experiment
Water	Chloroform	26.67	1.49 g mL^{-1}	0.5–64 mL min^{-1}	100 %	100 %

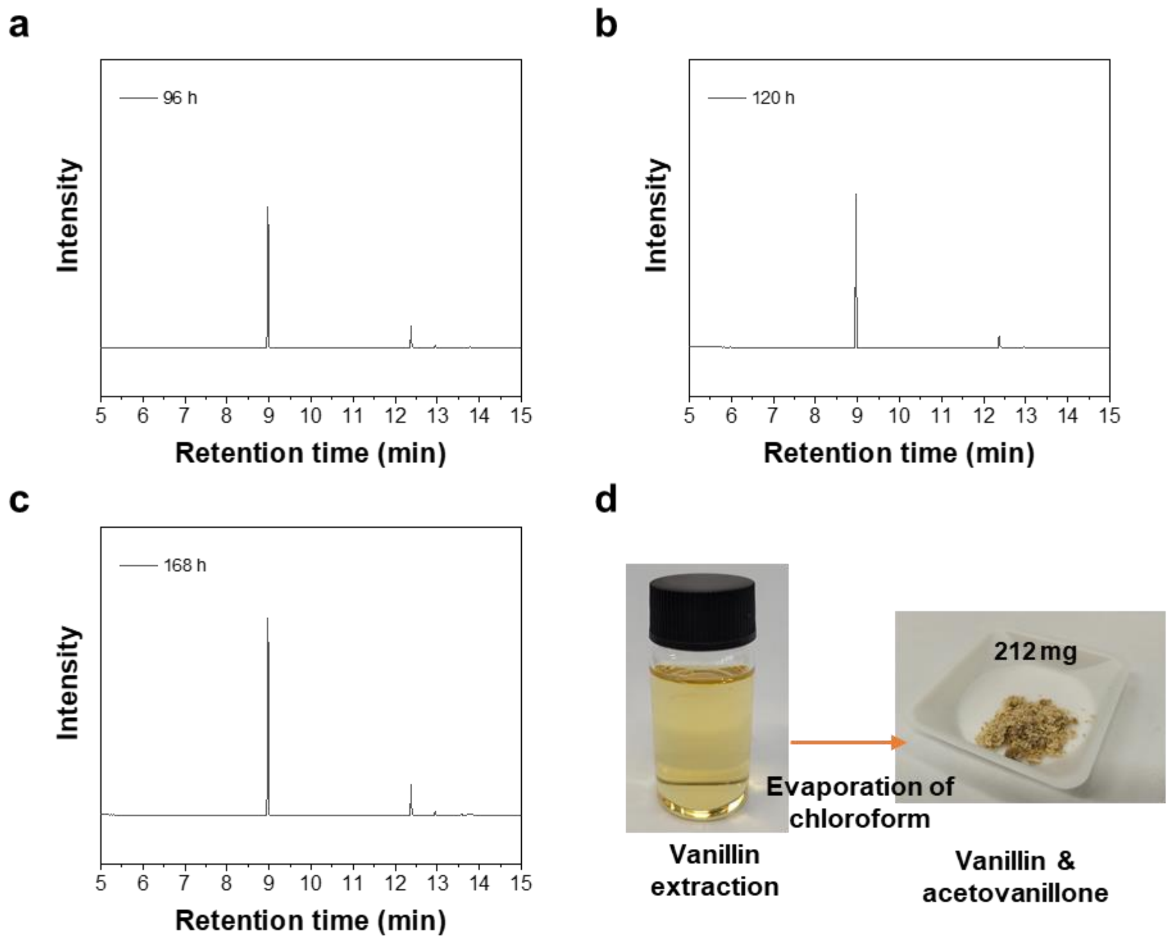

Figure S14. Enrichment and recovery of vanillin and acetovanillone from the mixture of PMA and lignin byproducts using DPT for a prolonged time of (a) 96 h, (b) 120 h, and (c) 168 h. (d) Photographs of the enriched vanillin and acetovanillone solution in chloroform and their powder after evaporation of chloroform.

4. Flow electrolyzers for low-voltage hydrogen production and PMA reactivation

4.1 Fabrication of the microchannel flow electrolyzer

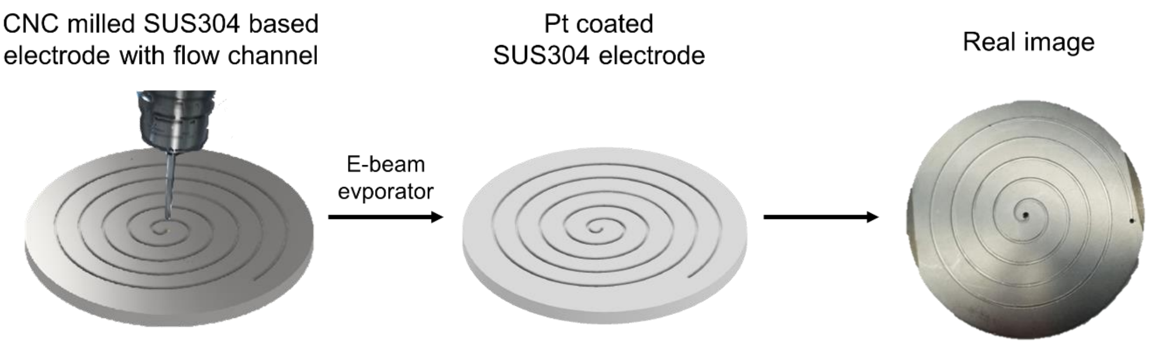

Figure S15. Schematic illustration for the fabrication of electrodes for flow electrolyzers.

4.2 Optimization of the microchannel flow electrolyzer

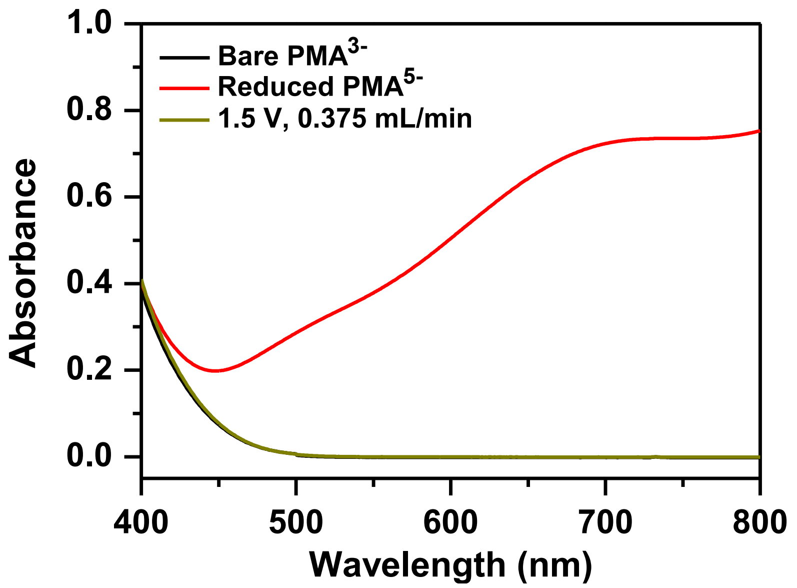

Figure S16. Complete re-oxidation of the reduced PMA (i.e., from PMA^{5-} to PMA^{3-}) via the microchannel flow electrolyzer, as confirmed by UV-vis absorbance spectroscopy. PMA^{5-} was re-oxidized at an applied voltage of 1.5 V and flow rate of 0.375 mL min^{-1} (residence time: 30 s). For comparison, the figure shows the absorbance spectra of the PMA without a reaction with lignin (i.e., bare PMA^{3-}).

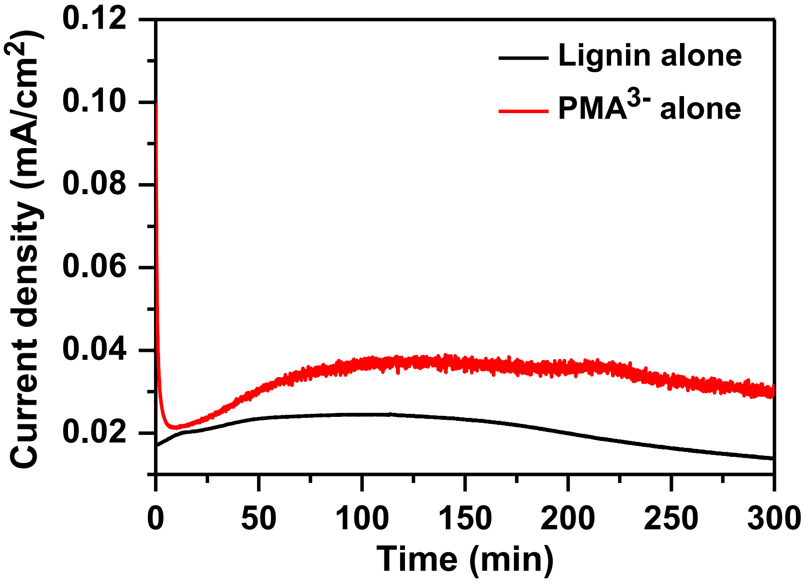

Figure S17. Chronoamperogram of the flow electrolyzer using non-reacted pristine PMA (i.e., PMA^{3-}) at 1.5 V. This result confirmed that electrons were efficiently extracted only from the reduced PMA^{5-}.

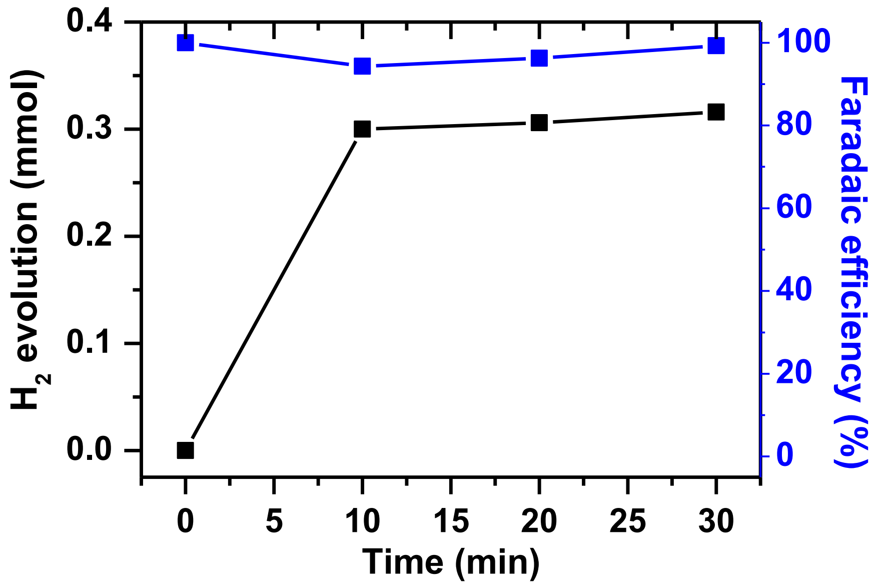

Figure S18. Hydrogen evolution and Faradaic efficiency profiles upon electrochemical reoxidation of the PMAs at 1.5 V and 0.375 mL min^{-1} (t^R: 30 s).

4.3 Flow vs. batch electrolyzers

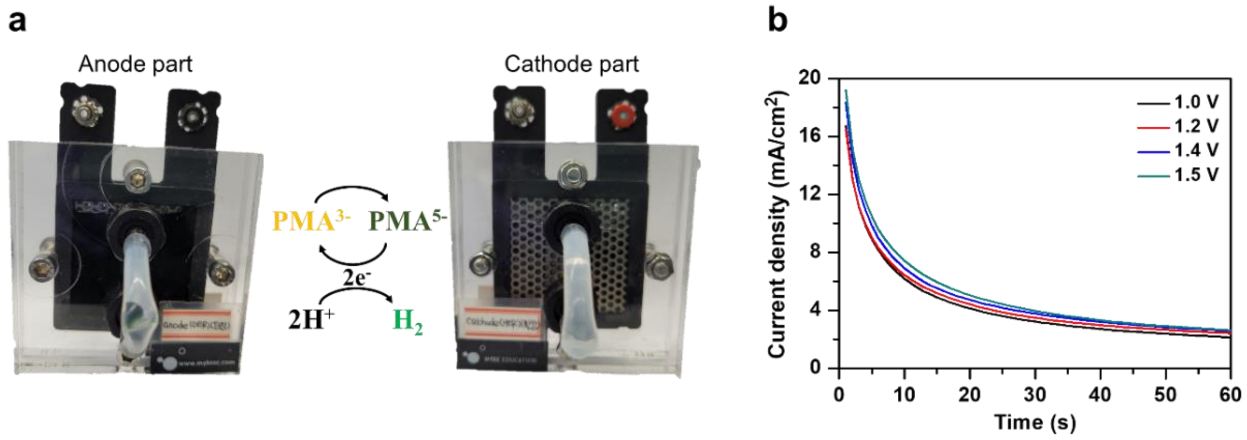

Figure S19. (a) Images of a batch-type electrolyzer based on a membrane-electrode assembly and (b) current density profiles during reoxidation of PMA^{5-} at various applied voltages. Current densities abruptly decreased due to the rapid consumption of the reduced PMA^{5-}, whose initial concentration was 0.5 M.

5. Integrated flow systems

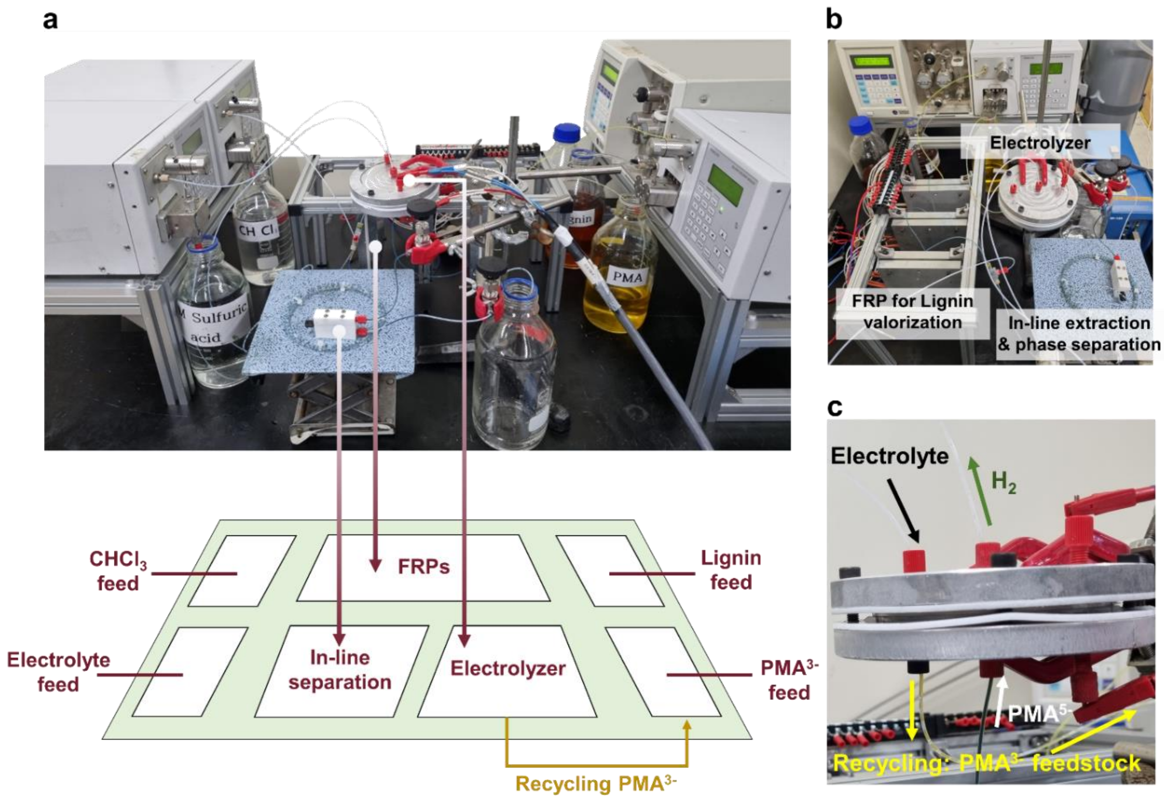

Figure S20. Integrated system for continuous-flow lignin valorization, byproduct separation, low-voltage hydrogen production, and PMA recycling. Photographs of (a, b) the integrated continuous-flow system and (c) electrolyzer component to re-oxidize the PMA^{5-} and generate hydrogen.

6. Potential effect of types of lignin on the lignin valorization

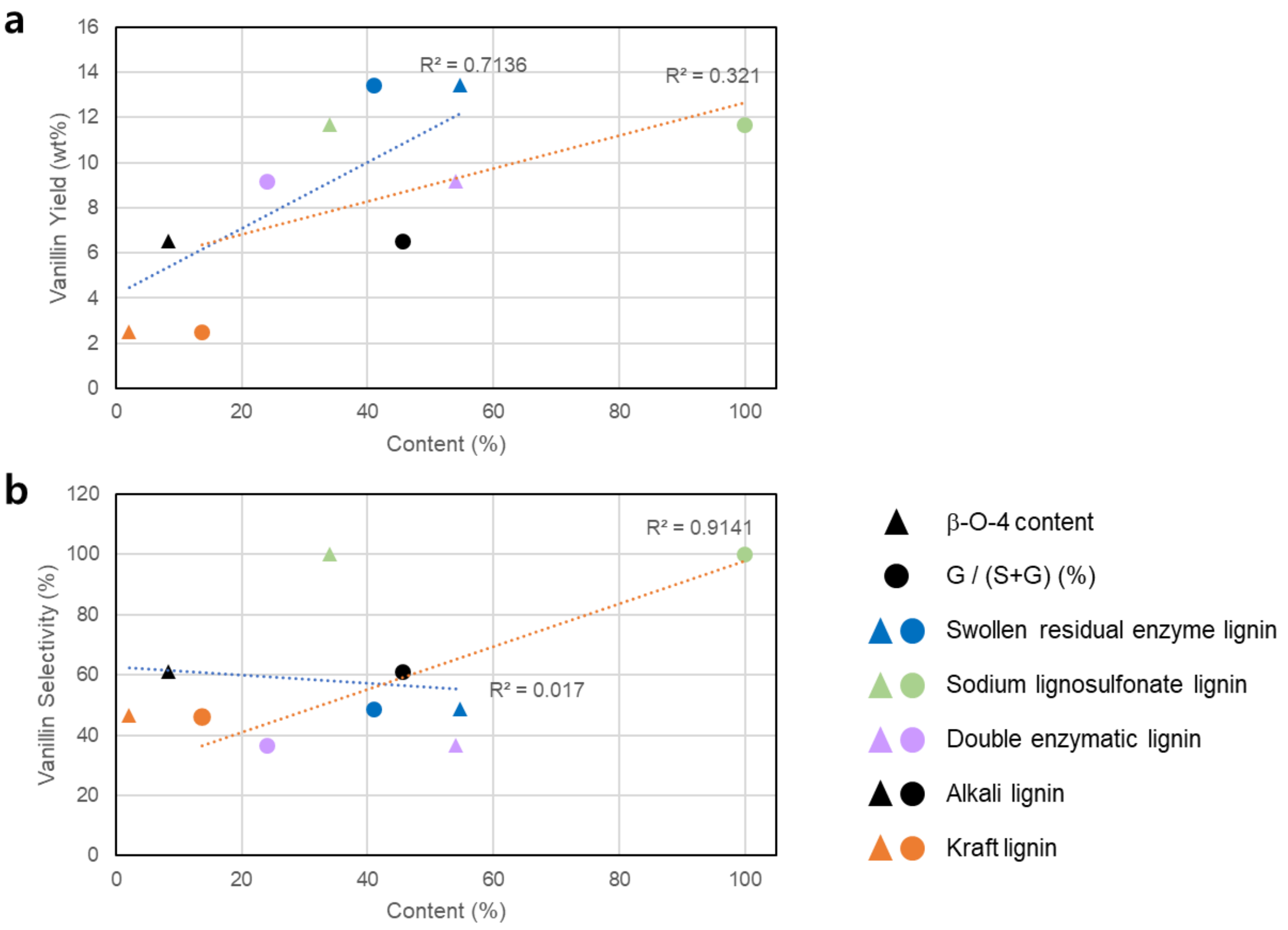

Figure S21. Relationship between the contents of β-O-4 linkages and the G / (S+G) ratio on (a) vanillin yield and on (b) vanillin selectivity. These graphs were drawn using the data[4] reported previously.

7. References

[1] C. Xu and T. Xie, *Ind. Eng. Chem. Res.*, 2017, **56**, 7593-7622.

[2] M. N. Kashid, Y. M. Harshe and D. W. Agar, *Ind. Eng. Chem. Res.*, 2007, **46**, 8420-8430.

[3] C. Noik, T. Palermo and C. Dalmazzone, *J. Dispers. Sci. Technol.*, 2013, **34**, 1029-1042.

[4] Y. Wang, S. Sun, F. Li, X. Cao, R. Sun, *Ind. Crops Prod.* **2018**, *116*, 116-121.
